# Social Cohesion of Functional Urban Areas (Example of Eastern Poland)

**DOI:** 10.1007/s11205-023-03119-4

**Published:** 2023-04-28

**Authors:** Anna Busłowska, Jacek Marcinkiewicz

**Affiliations:** Faculty of Economics and Finance, University of Białystok, Poland Warszawska Str., 63, 15-062 Białystok, Poland

**Keywords:** Social cohesion, Functional urban areas, Sigma convergence, O18, O52, R58

## Abstract

This paper is about studying social cohesion in functional urban areas (FUA). These territorial units become an important stakeholder and recipient of urban policy. Therefore, it is important to study problems of their development, including social cohesion. In the paper it is understood in spatial terms, i.e. it occurs when the differentiation of specific territorial units in terms of selected social indicators is reduced. In the research was studied the sigma convergence related to functional urban areas of vovodeship’s capital cities in five least-developed regions of Poland (so-called the Eastern Poland). The aim of this article is to investigate whether social cohesion is increased in the FUA of the Eastern Poland. The obtained results showed that only in three FUA in the analyzed period was sigma convergence but it was very slow process. In two FUA, no sigma convergence was identified. At the same time, it was observed that in all the analyzed areas there was an improvement in the social situation.

## Introdaction/Context

In the context of the functioning of the European Union (EU), the cohesion policy is the basic direction of the development of its regions. Territorrial-based approach has become the main development challenge, also in shaping urban policy, in particular within functional urban areas (FUA). In EU’s budgeting period 2014–2020 they became one of adressee of support in frame of a new instrument—Integrated Territiorial Investments (ITI). Therefore FUA’s research in the field of socio-economic processes, determinants and development problems is an important contribution to the scientific discussion and can provide important information for formulating goals and directions for further EU cohesion policy. In the current inclusive growth paradigm, it is particularly important to ensure social cohesion, which becomes the subject of political action at various levels. It is seen as a condition of political stability and security, as a source of prosperity and a necessary condition for improving the quality of life. At the same time, it should be noted, that defining and measuring social cohesion is quite broad and there is no consensus in this regard in the literature. This can be considered a disadvantage, but also an advantage. On the one hand, this diversity of approaches to social cohesion certainly limits the comparability of research carried out between different territorial units. On the other hand, it may allow for a better adjustment of research methods to the specificity of various territorial systems, that are the addressees of the development policy. An additional limitation in the scope of research is the availability of comparable data, especially at different administrative and territorial levels. Bearing in mind the above conditions and limitations, the authors want to join the discussion on the understanding and research of social cohesion, focusing on local systems, i.e. urban functional areas. It is a doubly difficult task, because there is not much research on the cohision of FUA in general (in Poland, e.g. Szafranek (2017) conducted such research regarding territorial cohesion), and there isn’t any specific methodological approach in this regard. Moreover, there are no standardized criteria for delimiting functional urban areas across the EU. This research focused on selected FUA in the Eastern Poland. Additionally, there is a difficulty in accessing extensive statistical data at the local level (in Poland, the FUA included municipalities, LAU level (Eurostat 1, 2022)). On the other hand, the approach to studying the social cohesion of these areas is an original research approach, in which the authors want to present possible ways of measuring the social cohesion of FUA based on available public data and proven statistical methods.

Therefore, the aim of this article is to study whether social cohesion is increased in the FUA of capital cities of the Eastern Poland regions (5 the poorest voivideships in Poland in terms of GDP per capita) by assessing the degree of sigma-type convergence. Selected research units are characterized by a territorial range, organizational structure and strategic documents programming their development.The authors would like to present the possibility of implementing sigma convergence indicator in the research of FUA’s cohesion (so far, in the literature, the measures of sigma convergence have rather focused on macrosystems). The research methodology also allowed to achieve an additional goal, that is: to determine the degree of social differentiation of the studied objects based on a synthetic measure of the social situation obtained through the use of the TOPSIS method. A research hypothesis was formulated that in FUA of capital cities of the Eastern Poland’s voivodeships (i.e. FUA Olsztyn, FUA Białystok, FUA Lublin, FUA Rzeszów and FUA Kielce) in the analyzed period of 2014–2021 was observed sigma convergence and social cohesion of these FUAs increased.

## Issues of Defining and Measuring Social Cohesion

The review of the literature on the subject shows, that social cohesion is a multidimensional concept. The way of presenting it, differs from the scientific perspective of researchers. It is also possible to distinguish some characteristic features of different definitions, that allow to combine them into specific groups. A large group of definitions related to a social cohesion concern various relations. Jenson (1998, p. 15–16) considered social cohesion as relationships in five dimensions: (1) Affiliation/isolation, (2) Insertion/exclusion, (3) Participation/passivity, (4) Acceptance/rejection, (5) Legitimacy/illegitimacy. Bernard (2000) distinguished formal and substantial forms of social cohesion in three spheres of human activity: economic, political and socio-cultural. Additionally Chan et al. (2006, p. 290) emphasized that these are "the vertical and the horizontal interactions among members of society as characterized by a set of attitudes and norms that includes trust, a sense of belonging and the willingness to participate and help, as well as their behavioral manifestations”. Thus they indicated, that the essence of these relations are norms and patterns of behavior. The norms and values as the foundations of social cohesion were also important in research of e.g.: Beck et al. (2001, p. 61), Berman and Phillips (2004, p. 2). They percived social cohesion as a “backgound for collective identities and the social world itself”, social cohesion is essencial for social quality (Berman & Phillips, 2004, p. 2). Social cohesion is also considered in terms of a network, the basis of which may be, for example, kinship and local voluntary organizations at communal level (Gough & Olofsson, 1999, pp. 63–84), or as a form of social solidarity^1^—a “complex set of solidarities—a network of networks” (Spiker, 2014, p. 95).

Social cohesion is also understood in terms of social inclusion, equal opportunities and equity for all citizens (Gough and Olofsson, 1999, p. 1–10; Acket et al., 2011, p. 3; OECED, 2012, p. 17; Spicker, 2014, p. 95; Bottoni, 2018, p. 838; Chauhan, 2021). This thread is also crucial in creating a cohesion policy within the EU, which is expressed in Article 3 of “The Treaty on European Union”. Morover in article 174 of “The Treaty on the Functioning of the European Union” it says, that social cohesion is necessary to promote overall harmonious development of European Union, in particular in terms of “reducing disparities between the levels of development of the various regions and the backwardness of the least favored regions". Building a high quality of life and social and economic well-being, as a necessary element is a common feature of many definitions of social cohesion (e.g. Chauhan, 2021, Dziembała, 2017, p. 143; Acket et al., 2011, p. 3; The Treaty on the Functioning of the European Union). An important element of such seen social cohesion, is the territorial approach, related to leveling out specific differences in development in individual regions. The EU cohesion policy, including urban approach, proposes polycentric development to counteract spatial imbalances. (Medeiros, 2019, p. 4; Faludi, 2006, p. 668). The spatial aspect in the understanding of social cohesion is very important, because the endogenous resources of the specific territory will determine its development opportunities and the quality of life improvement. In this sense, ensuring social cohesion will be associated with reducing disparities between the levels of development of the various regions. This theoretical approach was used in this paper.

Summing up above, it can be indicated, that rich theoretical background of social cohesion leads to its different classifications, divisions (e.g. Jeannotte, 2003, p. 3, Acket et.al., 2011, pp. 3–6; Schiefer & van der Noll, 2017, p. 586; Berman & Phillips, 2004, pp. 2–10). One of the most comprehensive and recent classifications of the definitions of social cohesion was presented by Schiefer and van der Noll (2017, p. 586). These authors indicate that social cohesion is perceived in three aspects: (1) relational (including social networks, participation, trust, tolerance), (2) ideal (responsibility for common good, solidarity, shared values) and (3) distributive (equality or inequality) objective and subjective quality of life. A review of the literature seems to confirm this division. The research in this paper is based on this third approach. Social cohesion is considered here by the spatial differentiation of the social situation (quality of life). The research hasn’t included areas such as norms and values, forms of relationships, solidarity, etc. They are difficult to study, and the data is often not available in open access.

Considering the issues of measuring cohesion, it can be assessed, that the method of measurement and the indicators used, are diverse. It is generally noted that measuring social cohesion is problematic and there is no standardized framework (Bottoni, 2018, p. 836; Spicker, 2014, p. 105). Bottoni (2018, p. 837) notes, that research on social cohesion is often limited to an endless list of social indicators without any conceptual analysis. Moreover, he emphasizes that, depending on the researched issues, organizations develop their own concepts of social cohesion. That author also draws attention to the persistent confusion between the factors influencing social cohesion and the components of social cohesion (Bottoni, 2018, p. 837).

In general, it can be indicated, that social cohesion studies are based both—on qualitative indicators, or on hard statistical data, or on two groups at the same time. Those approaches have both advantages and disadvantages. Relying on qualitative data may allow for carrying out extensive and in-depth analyses, but they are also time-consuming and burdened with high subjectivism. In the case of hard, quantitative indicators, the greatest limitation is obtaining of data. Their resources in official statistics may be limited, which can limit also a comparability, e.g. between different territorial units. A separate issue is also the appropriate selection of data for the research (Spicker, 2014, p. 105).

In studing social cohesion in terms of reducing disparities, the following key policy areas can be indicated: fiscal policy, employment and social protection, education, gender equality, migration, (OECED, 2012, p. 18–23; European Commission., 2022c. p. 135–186). In the EU approach of studing social cohesion, can be indicated cohesion reports and detailed indicators of social cohesion there presented. They are i.a.: the employment/unemployment rate, population at risk of poverty or social exclusion, population living in severe material deprivation, migrations, gender equality, participation of adults in education and training, early leavers from education or training, share of migrants relative to GDP per capita, difference between female and male employment rates, women in regional assemblies, Female Achievement Index (more: European Commision, 20222a, p. 135–186). To describe the EU’s social cohesion it is also used a synthetic indicator—the EU Regional Social Progress Index (EU-SPI). It is built on the approach of the global Social Progress Index, which can be understood as “the capacity of a society to meet the basic human needs of its citizens, establish the building blocks that allow people and communities to enhance and sustain the quality of their lives, and create the conditions for all individuals to reach their full potential”. The highest value of this index is in the regions (NUTS-2) of Western Europe, in the regions of central Europe, including Poland, the value of EU-SPI is weaker. In Poland, in most regions this index is at the level of 60–65, in four at the level of 55–60 and only in one at the level of 65–70 (European Commission, 2022c, pp. 181–183). It should be noted that the SPI index is a very useful^2^comparative tool at the level of NUTS-2 regions, but on a local scale its value is negligible, due to the lack of diverse, local-leveled, open access statistical data.

Having regard to the above, in this paper, the research study of social cohesion refers to the differentiation of social situations in selected FUAs included following areas of analysis: migration, labor market, including gender equality, education and social care. They therefore overlap with the areas mentioned in the EU cohesion reports, but not cover all of them. The selection of indicators used in the analysis (detailed further in the paper) was limited by the availability of public data at the level of local units (LAU, in Poland FUAs are made up of municipalities).

## Functional Urban Areas as Addressees of Cohesion Policy

The idea of the functional urban area, its specificity as a closed territorial system, is reflected in various theories of regional development for example theories based on the relationship between the central place and its adjacent area. It can be mentioned here core-periphery theory of J. Friedmann. He assumed the possibility of the development of the periphery, which depends on the core place, due to its greater potential and influence on the changes taking place in the entire area (Friedmann, 1967, 21–29). The functioning of FUA can be also related to the theory of polarized region of Boudeville (1966). Modifying Peroux's (1964) theory of growth poles, Boudeville pointed to the role of functional relations between the pole (e.g. the core of FUA) and its sphere of influence (e.g. other FUA units) (Darwent, 1969, 6–15; Szafranek, 2017, p. 116–117; Nowak, 2018, pp. 62–64).

In the current literature, the topic of functional urban areas, includes the study of their role in different socio-economic interactions, mainly through commuting between the central city and the rest of the functional zone (Karlsson & Olsson, 2006; Bode, 2008; Sykora & Mulicek, 2009; Śleszyński, 2014; Gu, et. al., 2015; Duranton, 2015; Hu & Han, 2019; Castells-Quintana, et.al., 2019). Due to the lack of standard criteria for delimiting FUA (Chen et.al. 2016), the definitions appearing in the literature are usually very general, intuitive. FUA is defined for example as urban geographical units consisting of various interrelated elements. These elements are rationally distributed in space and interconnected by various flows of materials, capital and information, and provide space for human living, production, recreation and business activities (Hu & Han, 2019, p. 2; Ferrão et.al., 2013, p. 6). In the context of the EU development policy, FUA can be defined as a continuous area which internal conditions are more conducive to development in relation to other places. Positive externalities, formal and informal institutions are more likely to emerge here. Moreover, in such areas, natural and cultural conditions and people’s preferences are more homogeneous, and human capital is characterized by greater synergy (Barca, 2009, p. 5). The joint EU—OECD definition, in the delimitation of FUA, is based on criteria such as population density, size and the daily mobility of inhabitants. (Dijkstra, et. al., 2019, p. 5).

The functional urban areas, as addressees of the cohesion policy, gained importance with the development of the place-based approach in the 2014–2020 EU programming period and introducing a new policy tool—Integrated Territorial Investment (Resolution EU 1303/2013, Article 36). The goal of ITI is to deliver cohesion policy in a territorially integrated way in order to increase its effectiveness and to deliver multi-dimensional and cross-sectoral interventions (Ferry, 2019, p. 5). Article 7 of the ERDF regulation (Regulation 1301/2013) indicated that sustainable urban development shall be undertaken through ITI and it was considered to handle territorial mismatch—the discrepancy between administrative and functional urban areas (Tosics, 2016; Miller&van der Zwet, 2018). In Poland, ITI was obligatorily implemented in all capital cities of voivodships and their functional areas, including five ones of the Eastern Poland (Olsztyn, Białystok, Lublin, Rzeszów and Kielce). General in Poland, delineating FUA’s around voivodship capital cities was based on the method of Śleszyński (2013, p. 179–182), that was using three types of criteria (indicators): (1) functional (number of people comuting to work, number of migration from FUA core (voivodship capital city); (2) socio-economic (share of people working in non-agricultural occupations, share of business entities, share of business entities classified in high-order services), (3) morphological (population density, share of completed dwellings). Finally FUAs covered the administrative boundaries of the municipalities, that met the criteria. In the Eastern Poland there are in total 58 such units in five functional area of capital cities. The smallest is FUA Olsztyn (with 7 municipalities and the biggest one is FUA Lublin with 16 municipalities (detailed in Table 2). Support of urban areas has become an important EU policy activity. It is therefore important to study the conditions of their development in order to better respond to their needs and pursue an effective policy in the future.

The Eastern Poland’s voivodships (NUTS-2 regions: warmińsko-mazurskie, podlaskie, lubelskie, podkarpackie and świętokrzyskie) are located at the EU's external border with Russia and Belarus and Ukraine. They are also specific regions in the context of cohesion policy, because after Poland's accession to the EU, they were the least developed EU regions. Now they are still one of the least developed areas, as in none of these regions GDP per capita exceeded 56% of the EU average (Table 1). Comparing to Poland's GDP per capita, these regions are also among the poorest in the country with a result of 71–74% of the national average. In addition, all the studied voivodeships are also characterized by a lower than the EU average (67), EU-SPI index, ranging from 58 up to 63 (detailed in Table 1). Moreover, in Poland since EU’s 2007–2013 programming period exists additional operational programme, that supports development of these 5 regions. It is therefore a macroregion with similar conditions and chalanges of development (the strategic areas of intervention).

**Table 1:** General data regarding the Eastern Poland voivodships (NUTS-2), 2020. *Source*: (European Commission, https, 2022b; Eurostat 2, https, 2022)

Regions	Warmińsko-mazurski	Podlaski	Lubelski	Podkarpacki	Świętokrzyski	EU-27
GDP, PPS per capita, in % of the EU27	53	56	52	52	54	100
GDP per capita, Poland = 100	71	74	69	69	73	n/a
EU-Social Progress Index	60	63	61	60	58	67

**Table 2:** General data regarding FUAs of voivodeship capital cities in the Eastern Poland, 2020. *Source*: (Statistics Poland, https, 2022)

Indicator/ FUA name	FUA Olsztyn	FUA Białystok	FUA Lublin	FUA Rzeszów	FUA Kielce	Poland
Name of voivodeship (NUTS-2 region)	Warmińsko-mazurski	Podlaski	Lubelski	Podkarpacki	Świętokrzyski	Total number of voivodeships in Poland: 16
Number of municipalities in FUA of voivodeship’s capital city	7	10	16	13	12	Total:348
						Average: 20
Density per 1 km^2^	162	244	346	359	252	FUA in Poland, average: 368
						Poland: 122
Population change per 1000 inhabitants	2.4	− 0.1	− 0.6	6.2	− 3.8	− 7.7 (before the pandemic in 2019 = -0.7)
Net internal migration per 1000 population	3.1	1.9	1.2	5.0	− 0.1	0.0
Average own income of municipality in FUA per capita in PLN	3796	3156	3110	3157	3127	3188
Registered unemployed persons per 100 inhabitants of working age	2.6	4.0	4.4	5.3	4.8	4.6

The analyzed functional areas don’t differ significantly in terms of the indicators selected in Table 2, but there can be observed some interesting conditions. Functional areas in the Eastern Poland are distinguished by a smaller number of units forming them, compared to the average amount in the country (Polish average: 20, the biggest FUA in Poland is Katowice-Gliwice (Silesia), which consists of 73 municipalities). The highest population density is in FUA Rzeszów (359) and in FUA Lublin (346). It is close to average density of all polish functional areas of voivodeship’s capitals (368). Whereas in FUA Olsztyn density is two times lower (162) and a little above then overall polish average (122). FUA Olsztyn is also characterized by the best social and economic situation among the other FUAs. It has the lowest rate of unemployed people per 100 people of working age (2.6, in Poland = 4.6) and the highest rate of municipality income per capita (3796 PLN, more then polish average = 3188 PLN). In other FUAs these indicators are close to the national average. Moreover the population change rate per 1,000 people is interesting. It is positive only in FUA Olsztyn (2.4, polish average = − 7.7, before the pandemic in 2019 = − 0.7) and Rzeszów (6.2). In the remaining FUAs in 2020, an outflow of population was recorded. The highest one was in FUA Kielce (− 3.8), the lowest in FUA Białystok (− 0.1). In all FUA, except for Kielce (− 0.1), the net internal migration per 1,000 people was positive. The highest was in FUA Rzeszów (5). It proves that these areas are poles of growth in the voivodship—an attractive place to live (Statistics Poland, https, 2022).

So where to place the social cohesion of the FUA? A territorial, spatial approach is necessary here. Cohesion of FUA, as recipients of EU policies, can be understood as levelling out development opportunities, reducing disparities within a given functional area. Therefore, in this paper social cohesion will be related to the reduction of disparities between studied municipalities in each functional area. The study is based on open access data available from Statistics Poland (https, 2022).

### Empirical Framework

In the literature on economic growth, in particular, we can find two meanings of the term of "convergence". The first, called sigma-convergence, concerns the reduction of dispersion (diversity) in living standards in different societies, while the second, called beta-convergence, occurs when poor countries develop faster than rich countries (Young, et al., 2008, p. 1083; Monfort, 2008, p. 3–5; Czasonis & Quinn, 2012, p. 183–204; Borsi & Metiu, 2015, p. 657–681). The subject of the research in this publication is sigma convergence. It was assumed that it occurs when the differentiation of the level of social development measured by the TOPSIS synthetic indicator (TOPSIS index) between individual units of FUA decreases in time. In general, changes in the variation of development levels can be measured by the standard deviation of the logarithms of the variable or by the coefficient of variation. In this publication, in order to verify the sigma convergence, a linear time series regression model was constructed for the value of the coefficient of variation according to the formula:

$$V_{t} = \alpha_{0} + \alpha_{1} t + \varepsilon_{t}$$

where: ⍺_0_, α_1_—model parameters, ε_*t*_—random component of the equation, t—time variable (t = 2014, …, 2021).

Sigma convergence occurs when the slope of the trend line, i.e. the rating of the parameter α_1_, is negative and statistically significant. The obtained results were presented below in a graphical and tabular form.

The research process consisted of 2 main stages. Firstly the TOPSIS method was used to construct synthetic indices of the diversity of social situation in municipalities of functional urban areas in the Eastern Poland based on selected variables. In the next stage, based on these TOPSIS indices, coefficients of variation in each FUA were calculated in order to evaluate sigma convergence. The research period covered the years 2014–2021. The research procedure included the following steps: (Hwang & Yoon, 1981; Roszkowska & Filipowicz-Chomko, 2020, pp. 1219–1241, Marcinkiewicz&Matel 2017, pp. 170–182):

Coefficients of variation in individual years and for individual studied objects (FUA) were determined on the basis of the differentiation of the level of the social situation (TOPSIS index) in following thematic areas: migration, education, social support, labor market (including context of gender equality), which is compatible with the EU approach. Therefore, at the beginning in each FUA, the following variables were selected to the research:
  X_1_—total net migration per 1000 population,
  X_2_—beneficiaries od social assistance per 10 thousand inhabitants,
  X_3_—households benefiting from community social assistance according to criterion of income,
  X_4_—share of the registered unemployed persons in the population in the working age,
  X_5_—employed persons per 1000 total population,
  X_6_—equality index in access to the labor market (with the variable rescaled to stimulus, with the optimal value 0.5—each deviation of the index from the value of 0.5 is treated by the authors as a deviation from equality of access to the labor market),
  X_7_—gross enrollment rate.
  All variables come from the local database of Statistics Poland. Selection of variables depended on their availability in official, public statistics at the local level (LAU, municipalities). All the variables used in the study are relative in nature, which allowed for the creation of a cumulative index and comparability of the examined functional urban areas in terms of time and space. The variables used in the study were selected in terms of content (so that they indicate significant changes in the areas describing social cohesion) and statistical (so that they are characterized by sufficiently high differentiation and mutually low correlation coefficients—so that they don’t duplicate the same information).
2. Establishing a list of potential explanatory variables and determining their nature, indicating stimulants and distimulants. Variables X_2_ , X_3_ , and X_4_ were recognized as distimulants and the rest as stimulants.
3. Normalizing of explanatory variables according to the formula of zero-unitarization:
  (a) For stimulants:
  $${z}_{ikt}= \frac{{x}_{ikt}- \underset{i}{\mathrm{min}}\left\{{x}_{ikt}\right\}}{\underset{i}{\mathrm{max}}\left\{{x}_{ikt}\right\}- \underset{i}{\mathrm{min}}\left\{{x}_{ikt}\right\}}$$
  (b) For distimulants:
  $${z}_{ikt}= \frac{{\underset{i}{\mathrm{max}}\left\{{x}_{ikt}\right\}- x}_{ikt}}{\underset{i}{\mathrm{max}}\left\{{x}_{ikt}\right\}- \underset{i}{\mathrm{min}}\left\{{x}_{ikt}\right\}}$$
  where: $$i$$- municipality ($$i=\mathrm{1,2}, \dots ,n)$$; $$k$$- number of the variable ($$k=\mathrm{1,2}, \dots ,7$$), $$t$$- year ($$t=2014-2021)$$; $$\underset{i}{\mathrm{max}}\left\{{x}_{ikt}\right\}$$- maximum value of the k in the investigated year; $$\underset{i}{\mathrm{min}}\left\{{x}_{ikt}\right\}$$- minimum value of the *k* in the investigated year.
  For each of the functional areas (due to their specificity), their own development patterns were selected based on data from the entire research period.^3^ An important issue is assigning weights to individual variables. For the purposes of this study, constant weights were used, meaning that for each variable defining the partial structure, the same meaning was given. The use of other weights would require more in-depth substantive analysis, relying on the expert method or on the possessed statistical material. In this case, finding objective criteria would be difficult.
4. TOPSIS Index:^4^
  Calculation of the Euclidean distance of municipalities from the pattern of development ${z}^{+}= \left(\mathrm{1,1}, \dots ,1\right)$ and anti-pattern of development ${z}^{-}= \left(\mathrm{0,0}, \dots ,0\right)$:
  $${d}_{it}^{+}= \sqrt{{\sum }_{k=1}^{m}{( {z}_{ikt} - {z}_{k}^{+} )}^{ 2}}$$
  $${d}_{it}^{-}= \sqrt{{\sum }_{k=1}^{m}{( {z}_{ikt} - {z}_{k}^{-} )}^{ 2}}$$
  Then, the values of the synthetic measures of social situation for each municipality and year were calculated:
  $${q}_{it}= \frac{{d}_{it}^{-}}{{d}_{it}^{-}+ {d}_{it}^{+}}$$
  ${d}_{it}^{-}$- distance from the anti-pattern of development for the $i$ municipality,
  ${d}_{it}^{+}$- distance from the pattern of development for the $i$ municipality,
  ${q}_{it}$– value of the synthetic measure for the $i$ municipality (TOPSIS index).
5. In the 2nd stage of the study, on the basis of the obtained synthetic TOPSIS index determining the level of the social situation for each of the municipality of analyzed FUA in the studied years, the coefficient of variation (V_t_ ) was calculated and time series regression model was developed. Moreover, TOPSIS indexes allowed to assess the level of differentiation of the social situation in individual FUA units. The results were presented in a graphic and descriptive form below.

### Empirical Results

The following research results are presented separately for each of the functional area, that was tested. Comparing the areas with each other wouldn’t be correct due to different development patterns/anti-patterns in each FUA according to TOPSIS procedure. However, the study research allows for drawing generalized conclusions about the convergence process taking place in studied functional areas, its intensity, as well as general diversity of social situation. The research period covered 8 years, i.e. 2014–2021. To keep the presentation of empirical results more transparent, only the beginning and end of this research period were characterized in presentation of TOPSIS results (stage 1 of research process).

#### FUA Olsztyn

The average value of the synthetic TOPSIS index in FUA Olsztyn in 2021 ranged from 0.410 to 0.651 (Table 3). This syntehtic index was gradually increasing until 2019 and since 2020 in most municipalitiesit has decreased, probably due to the COVID-19 pandemic (it has also been observed in other FUAs). The dynamics of changes of the TOPSIS indices in the analyzed period 2014–2021, in municipalities of FUA Olsztyn was diversified, from 6% up to 70%. In the dynamics of the TOPSIS index, it is clear, that the rate of change was quite low in the core city of FUA, Olsztyn (13%). Most of other territorial units (rural and urban–rural municipalities (having at least 1 town within their border) recorded much higher dynamic. The biggest change however, was in the units with the lowest TOPSIS index in 2014 (Purda, Barczewo). However, despite the high dynamics, the position in the ranking of researched units has hardly changed. However, a decrease of coefficient of variation (from 3.12 in 2014 to 0.159 in 2021) indicates a reduction of social disparity in this FUA.

**Table 3:** TOPSIS index in FUA Olsztyn (2014, 2021). Source: own elaboration

Name of municipality/Year	2014	Position, 2014	2021	Position, 2021	Change, 2014 = 100
Barczewo (3*)	0.275	6	0.466	5	169
Dywity (2)	0.388	3	0.504	3	130
Gietrzwałd (2)	0.377	4	0.485	4	129
Jonkowo (2)	0.294	5	0.410	7	139
Purda (2)	0.256	7	0.434	6	170
Stawiguda (2)	0.559	2	0.594	2	106
Olsztyn (1)	0.574	1	0.651	1	113
Arithmetic mean	0.389	n/a	0.506	n/a	n/a
Standard deviation	0.121	n/a	0.081	n/a	n/a
Coefficient of variation	0.312	n/a	0.159	n/a	n/a

^*^Type of municipalities (gminas): 1- urban, 2- rural, 3-urban–rural

**Fig. 1:**
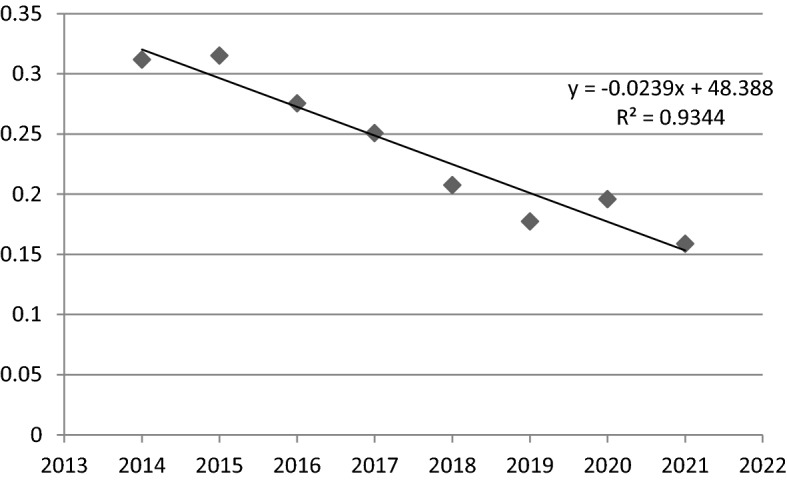
A linear time regression model for the coefficient of variation values in FUA Olsztyn (2014–2021). *Source*: own elaboration

Moreover, considering the linear time regression model for the value of the coefficient of variation V_t_ (Fig. 1), it can be concluded, that sigma convergence was observed in this functional area. This is indicated by the value of the parameter α_1_ (− 0.0239) and the negative slope of trend line. In general, the level of convergence is very slow, because with the passage of time by one unit (here: one year) the sigma convergence in the studied area was increasing only by 0.0239 units. The statistical significance of the above model is confirmed by the results of the sigma convergence regression model parameters estimation (Table 4). Quite high value of the R^2^ (0.934) and the p-value (clearly below 0.05) indicate, that the model is statistical significant.

**Table 4:** The results of the estimation of the parameters of the sigma convergence linear time regression model in FUA Olsztyn. *Source*: own elaboration

Name	R^2^	Coefficient	Standard error	*p*-value
The variable t	0.9344	− 0.0239	0.0026	0,00,009

In conclusion it can be highlighted, that in all units in FUA Olsztyn their social situation improved, but the dynamics of these changes was very different. It was also found, that a sigma convergence was taking place, although it was very slow. However it can be generally assessed positively and it can be said, that social cohesion in FUA Olsztyn in the analyzed period was increasing.

#### FUA Białystok

The average value of the synthetic TOPSIS index in FUA Białystok in 2021 ranged from 0.447 to 0.639 (Table 5). The change of the TOPSIS index in the analyzed period was much less diversified than in e.g. FUA Olsztyn. The changes were in range of 14–57%. Changes in the social situation in the municipalities of this FUA were quite even (mostly ranged from 27 to 38%). The smallest improvement was recorded in the core city–Białystok (14%), which in 2021 wasn’t ranked 1st. This indicates that other municipalities are catching up with social disparities. This is also evidenced by the slight decrease of coefficient of variation (from 0.137 to 0.115).

**Table 5:** TOPSIS index in FUA Białystok (2014, 2021). *Source*: own elaboration

Name of municipality/Year	2014	Position, 2014	2021	Position, 2021	Change, 2014 = 100
Choroszcz (3)	0.494	2	0.639	1	129
Czarna Białostocka (3)	0.366	9	0.500	8	136
Dobrzyniewo Duże (2)	0.455	4	0.577	6	127
Juchnowiec Kościelny (2)	0.467	3	0.597	4	128
Łapy (3)	0.324	10	0.447	10	138
Supraśl (3)	0.397	8	0.622	2	157
Turośń Kościelna (2)	0.434	6	0.563	7	130
Wasilków (3)	0.443	5	0.592	5	134
Zabłudów (3)	0.402	7	0.464	9	115
Białystok (1)	0.538	1	0.615	3	114
Arithmetic mean	0.432	n/a	0.561	n/a	n/a
Standard deviation	0.059	n/a	0.064	n/a	n/a
Coefficient of variation	0.137	n/a	0.115	n/a	n/a

**Fig. 2:**
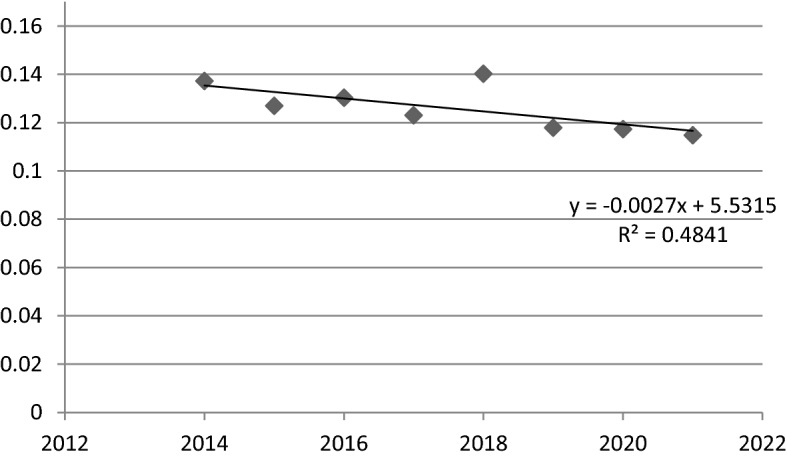
A linear time regression model for the coefficient of variation values in FUA Białystok (2014–2021). *Source*: own elaboration

Considering the linear time regression model for the value of the coefficient of variation (Fig. 2) and the results of model parameters estimation (Table 6), it can be noticed, that in FUA Białystok sigma convergence hasn’t been decisively observed. Despite the negative value of the parameter α_1_ (− 0.0027) and slightly negative slope of the trend line, the results of estimation of model parameters indicate rather its statistical insignificance. This conclusion is confirmed by the low value of the R^2^ (0.484) and the p-value (more then 0.05).

**Table 6:** The results of the estimation of the parameters of the sigma convergence linear time regression model in FUA Białystok. *Source*: own elaboration

Name	R^2^	Coefficient	Standard error	*p*-value
The variable t	0.4841	− 0.0027	0.0011	0.0553

In conclusion, it should be stated, that in FUA Białystok, in all studied units the social situation improved. Moreover, the sigma convergence due to results of estimation of model parameter wasn’t decisively observed. However, in FUA Bialystok in 2021 it was noticed a rather small coefficient of variation (11.5%), which may indicate a quite similar social situation between municipalities in this FUA.

#### FUA Lublin

The average value of the TOPSIS index in FUA Lublin in 2021 ranged from 0.409 to 0.665 (Table 7). The dynamics of changes this index, compared to 2014, was quite similar in most researched units (from 15 to 30%) and only in four units it ranged from 44 to 66%. It shows rather the steady rate of improving of social situation in most units. General, the highest changes were in all rural municipalities. However, the positions in the ranking between 2014 and 2021 didn’t change significantly. In general, the decrease in differentiation in this FUA is proved by the decline of coefficient of variation, which in 2021 was 12.6%.

**Table 7:** TOPSIS index in FUA Lublin (2014, 2021). *Source*: own elaboration

Name of municipality/Year	2014	Position, 2014	2021	Position, 2021	Change, 2014 = 100
Lubartów (1)	0.511	3	0.6072	2	119
Lubartów (2)	0.293	14	0.449	14	153
Głusk (2)	0.498	4	0.591	4	119
Jabłonna (2)	0.391	12	0.510	12	130
Jastków (2)	0.421	9	0.549	6	130
Konopnica (2)	0.458	6	0.529	9	115
Niedrzwica Duża (2)	0.433	8	0.539	7	125
Niemce (2)	0.470	5	0.593	5	126
Strzyżewice (2)	0.353	13	0.515	10	146
Wólka (2)	0.4133	11	0.500	13	121
Spiczyn (2)	0.257	16	0.425	15	166
Nałęczów (3)	0.436	7	0.533	8	122
Świdnik (1)	0.518	2	0.6069	3	117
Mełgiew (2)	0.289	15	0.409	16	142
Piaski (3)	0.4134	10	0.515	11	125
Lublin (1)	0.555	1	0.665	1	120
Arithmetic mean	0.419	n/a	0.534	n/a	n/a
Standard deviation	0.084	n/a	0.067	n/a	n/a
Coefficient of variation	0.200	n/a	0.126	n/a	n/a

**Fig. 3:**
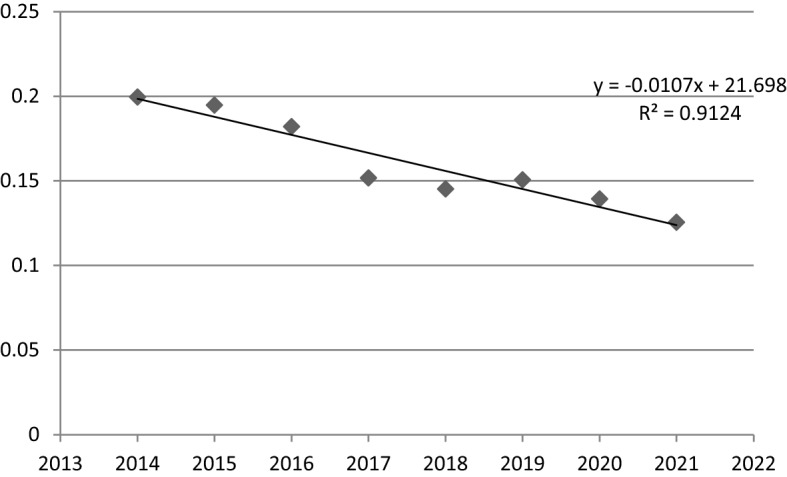
A linear time regression model for the coefficient of variation values in FUA Lublin (2014–2021). *Source*: own elaboration

Moreover, taking into account the linear time regression model for the value of the coefficient of variation (Fig. 3), it can be observed that sigma convergence in FUA Lublin was taking place. This is proved by the value of the parameter α_1_ (-0.0107) and negative slope of trend line. However, also in this case, the convergance progress is even slower, then in FUA Olsztyn. Social cohesion was increasing only by 0.0107 units per year. The statistical significance of the above model is confirmed by the results of the sigma convergence regression model parameters estimation (Table 8), i.e. a very high value of R^2^ (0.9124) and very low *p*-value (0.0002).

**Table 8:** The results of the estimation of the parameters of the sigma convergence linear time regression model in FUA Lublin. *Source*: own elaboration

Name	R^2^	Coefficient	Standard error	*p*-value
The variable t	0.9124	− 0.0107	0.0014	0.0002

In conclusion, it should be highlighted, that a slow sigma-type convergence in FUA Lublin has been observed. Nevertheless, it can be assessed, that social cohesion has improved here in the analyzed period. Moreover, TOPSIS indices show, that all units have improved their social situation, with the dynamics of change being quite similar in most municipalities. The rate of change was high in rural municipalities, lower in urban and urban–rural areas.

#### FUA Rzeszów

The average value of the TOPSIS indexes in FUA Rzeszów in 2021 ranged from 0.450 to 0.778 (Table 9). The dynamic of changes in analyzed period was between 14 and 58%. It was high in all types of municipalities. The high rate of change in this case was also observed in the central city of Rzeszów (33%, e.g. in FUA Olsztyn and Białystok in capital city it was one of the weakest). The high pace of changes, improved the results in the ranking of 4 municipalities in this FUA. In addition, the coefficient of variation (14.4% in 2021) is informing about a rather small differentiation of municipalities in this FUA.

**Table 9:** TOPSIS index in FUA Rzeszów (2014, 2021). *Source*: own elaboration

Name of municipality/Year	2014	Position, 2014	2021	Position, 2021	Change, 2014 = 100
Łańcut (1)	0.564	2	0.706	2	125
Czarna (2)	0.394	9	0.546	9	139
Łańcut (2)	0.445	6	0.525	11	118
Boguchwała (3)	0.507	4	0.579	8	114
Chmielnik (2)	0.377	12	0.595	5	158
Głogów Małopolski (3)	0.417	8	0.5935	7	142
Krasne (2)	0.511	3	0.633	4	124
Lubenia (2)	0.331	13	0.450	13	136
Świlcza (2)	0.419	7	0.529	10	126
Trzebownisko (2)	0.495	5	0.654	3	132
Tyczyn (3)	0.382	10	0.5941	6	156
Czudec (2)	0.380	11	0.490	12	129
Rzeszów (1)	0.587	1	0.778	1	133
Arithmetic mean	0.447	n/a	0.590	n/a	n/a
Standard deviation	0.076	n/a	0.085	n/a	n/a
Coefficient of variation	0.170	n/a	0.144	n/a	n/a

**Fig. 4:**
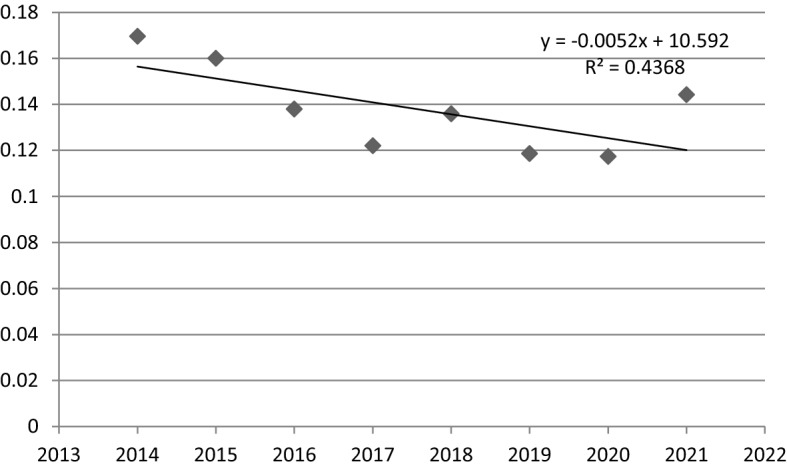
A linear time regression model for the coefficient of variation values in FUA Rzeszów (2014–2021). Source: own elaboration

Taking into account the linear time regression model for the coefficient of variation (Fig. 4) and the results of the sigma convergence regression model parameters estimation (Table 10), it can be noticed, that in FUA Rzeszów sigma convergence couldn’t be decisively observed. Despite the negative value of the parameter α_1_ (− 0.0052) and negative slope of the trend line, the results of estimation of model parameters indicate its statistical insignificance. This is also confirmed by the quite low value of the R^2^ (0.437) and the high *p*-value (0.074).

**Table 10:** The results of the estimation of the parameters of the sigma convergence linear time regression model in FUA Rzeszów. *Source*: own elaboration

Name	R^2^	Coefficient	Standard error	*p*-value
The variable t	0.4368	-0.0052	0.0024	0.0744

To conclude, the study indicates that in FUA Rzeszów sigma convergence can’t be definitely confirmed. In general, however, it can be noted that all territorial units have improved their social situation expressed in the growth of TOPSIS indices. Moreover the coefficient of variation in research period has been decreased, which proves a lower diversity of the studied units.

#### FUA Kielce

The average value of the TOPSIS index in FUA Kielce in 2021 ranged from 0.474 up to 0.723 (Table 11). In this functional area, the dynamics of change was very stretched, from 13% up to 90%. A general improvement in the social situation was recorded mainly in rural (18-56%), as well as in urban–rural municipalities (17-90%). Also in this case, despite a significant changes in some municipalities, only 4 of them improved their positions in the ranking. The core city–Kielce, had the weakest dynamic of growth (13%) and at the beginning and at end of the research period wasn’t ranked 1st. Furthermore the coefficient of variation indicates, that the differentiation of the social situation decreased gradually from 22.3 to 11.3%.

**Table 11:** TOPSIS index in FUA Kielce (2014, 2021). *Source*: own elaboration

Name of municipality/Year	2014	Position, 2014	2021	Position, 2021	Change, 2014 = 100
Chęciny (3)	0.430	6	0.540	11	126
Chmielnik (3)	0.250	12	0.474	12	190
Daleszyce (3)	0.382	9	0.557	7	146
Górno (2)	0.361	11	0.544	10	151
Masłów (2)	0.470	4	0.554	8	118
Miedziana Góra (2)	0.379	10	0.592	4	156
Morawica (3)	0.616	1	0.723	1	117
Piekoszów (2)	0.436	5	0.575	5	132
Nowiny (2)	0.535	3	0.688	2	129
Strawczyn (2)	0.383	8	0.565	6	147
Zagnańsk (2)	0.387	7	0.546	9	141
Kielce (1)	0.565	2	0.637	3	113
Arithmetic mean	0.433	n/a	0.583	n/a	n/a
Standard deviation	0.096	n/a	0.066	n/a	n/a
Coefficient of variation	0.223	n/a	0.113	n/a	n/a

**Fig. 5:**
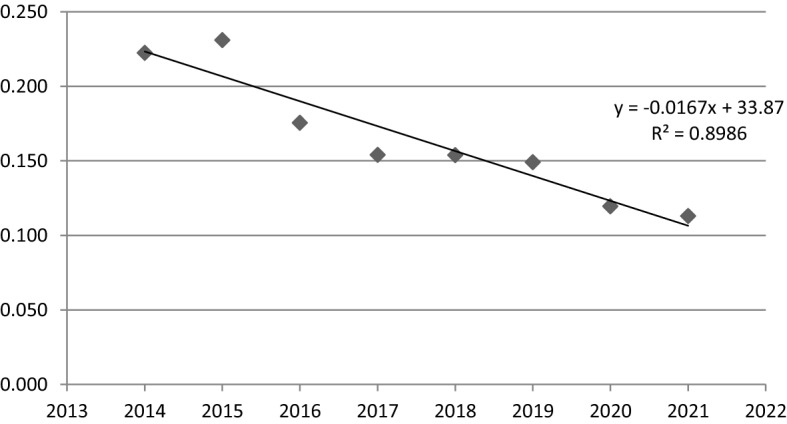
A linear time regression model for the coefficient of variation values in FUA Kielce (2014–2021). *Source*: own elaboration

Regarding the linear time regression model for the value of the coefficient of variation (Fig. 5) and the results of estimation of the sigma convergence regression model parameters (Table 12), it can be noticed, that sigma convergence in FUA Kielce in 2014–2021 has been observed. It is confirmed by the value of the parameter α_1_ (− 0.0167) and negative slope of the trend line. Statistical significance is proved by high R^2^ (0.899) and low *p*-value (only 0.0003). In this case, it can therefore be stated, that the social cohesion was improveing by 0.0167 units per year.

**Table 12:** The results of the estimation of the parameters of the sigma convergence linear time regression model in FUA Kielce. *Source*: own elaboration

Name	R^2^	Coefficient	Standard error	*p*-value
The variable t	0.8986	− 0.0167	0.0023	0.0003

To sum up, it can be highlighted, that in all municipalities of FUA Kielce social situation has improved, but the dynamics of these changes was very diverse. Moreover, sigma convergence was observed here and it can be assessed, that in the analyzed period the social cohesion of FUA Kielce increased.

In general, referring to the results of the above study of five FUAs of the Eastern Poland, the following conclusions can be highlighted:

- The obtained sigma convergence indicators together with results of estimation of the linear time regression model parameters, indicate that social cohesion has been observed in 3 out of 5 analyzed FUAs, i.e. in: Olsztyn, Lublin, and Kielce. The level of sigma convergence in these functional urban areas was rather slow, not exceeding 0.03 units per year, with the highest sigma convergence in FUA Olsztyn (0.0239) and the lowest in FUA Lublin (0.0107). Therefore it can be assessed that in general social cohesion has increased in these FUAs.
- In FUA Białystok and Rzeszów the results of model parameters estimation indicate their statistical insignificance. Therefore it isn’t possible to assess with certainty, whether convergence in analyzed period was taking place there. In these areas, however, was observed a decreasing level of coefficient of variation. Thus, it can be assumed, that the differentiation between units in those FUAs was also getting smaller in analyzed period. Possibly, it may indicate better social cohision in these FUAs.
- Referring to the TOPSIS indices, a systematic improvement of social situation was observed in all FUAs in analyzed period. The dynamics varied in the studied areas. Nevertheless since 2020, a decreases in the dynamics of the TOPSIS index was observed in many municipalities in researched FUAs. Undoubtedly, it was related to the COVID-19 pandemic, that negatively affected the functioning of the economy and the society in moast of the countries in the world.
- Regarding to the TOPSIS indices, it was also noted that significant improvements in the social situation occurred mainly in rural municipalities, whose social situation at the beginning of the research period was mostly the worst. In case of units with the lowest TOPSIS index values, the growth dynamics was the highest. This may confirm, that the policy of supporting less developed areas brings benefits. In the EU’s 2014–2020 programming period, all these FUAs used the ITI instrument. The implemented projects concerned i.a. education, social and health care, increasing employment support.
- Moreover, in the FUA Lublin and Olsztyn, a definite predominant position of the central city of the functional area was observed in comparison to the other municipalities. This aspect can be read as negative, showing a gap in the social situation between the core city and other units. In other functional areas the capital city was ranked high, but didn’t have a decisive advantage over other units. Only in Białystok at the beginning of the research period, greater differences were observed, which, however, decreased by 2021. Besides the dynamics of improving the social situation in capital cities was one of the weakest of researched units (except Lublin and Rzeszów, where the pace of change was quite high).

Generally, therefore, the hypothesis can be positively verified only in 3 FUA Olsztyn, Lublin and Kilece. No sigma convergence was observed in the other two FUAs. However, the decrease in the coefficient of variation in all studied functional areas in the period 2014–2021 shows that social differences decreased in each of them. This proves that the conditions of development are becoming more similar, which improves a living conditions and quality of life in each of FUA.

## Conclutions

Social cohesion is a multi-dimensional concept, which is difficult to research. There are many methodological approaches, which are often dependent on the type of research studies. One way is to study sigma convergence. In general it concerns in particular the economic situation of countries or regions. In this paper the sigma convergence was used to taste social cohesion of FUAs. In the literature review, the authors did not find such ways of examining social cohesion in relation to FUA. This is, therefore, a new contribution to the discussion regarding the study of social cohesion in general, as well as in FUAs. In the proposed research approach, various data regarding social cohesion can be included in the construction of a synthetic indicator and tasting sigma convergance. However, one of significant limitation, which occurred in the case of this research study, was the availability of data in such specific units as FUAs. Data at the local level in open databases is much poorer than e.g. at the regional or national level.

Nevertheless, urban areas are an important subject of development policy, and the study of FUA in the context of EU cohesion policy is becoming more intensive and justified. The studies on FUA’s cohesion available in the literature review concern in particular the territorial cohesion. It is understood broadly as a reduction in inequality in the economic, social, environmental and spatial spheres, which makes a social cohesion one of elementof territorial cohesion. Extensive research in this subject is carried out, e.g. by ESPON (European Observation Network for Territorial Development and Cohesion). The results of studing cohesion in frame of social indicators (inclusion and quality of life) show a general trend towards cohesion (ESPON, https, 2022). Moreover, with regard to the studies of social cohesion, it seems interesting the EU-SPI index approach. In a multidimensional way, it illustrates the social progress in EU regions and countries. This index higlightes, that the regions of the Eastern Poland are among the least developed. This shows the need to continue support of social development. This also applies to FUA.

The research study showed a diverse social situation of FUAs of capital cities in the Eastern Poland. However, in most of them, a slow rate of sigma convergence was observed and in all FUAs the differentiation expressed by the coefficient of variation decreased. This proves that some processes of coherence are taking place there. Therefore continuation of the development policy, especially in social sphere is still necessary. The authors are therefore of the same opinion as, e.g., Szafranek (2017, p.126), who claims that FUAs and the related policy can help the cohesion only, if conditions for the diffusion processes are created. In general, EU cohesion policy, including urban cohesion policy, should assume greater polycentrism of development aimed at counteracting diversification (e.g. in the Eastern Poland mainly in the case of FUA Olsztyn and Lublin in order to prevent the deepening of differences between the central (capital) city and other parts of the functional area). The basis for polycentric development should be the use of internal resources and networking.

## Footnotes

1 In this case, he understood solidarity as a principle of mutual responsibility and in part to a sense of shared identity and commitment (Spiker, 2014, p. 95).

2 This index consists of 55 partial indicetors, incl. in the following areas i.a.: nutrition and basic medical care, Water and sanitation, Shelter, Personal security, Access to basic knowledge, Health and wellness, etc. Unit of measurement: 0–100 scores. (more: European Commission. 2022c, p. 185).

3 It should be borne in mind that in the literature on the subject there are other ways of normalizing the variables: quotient transformation, standardization, ranking of variable values. In this article, normalization with the use of the unitarization formula was conducted, where after transformation the variables have the same range of variability (the interval of the transformed variable is equal to unity).

4 The TOPSIS method (Technique for Order Preference by Similarity to anIdeal Solution), proposed by Hwang & Yoon (1981), is one of the methods of linear ordering of multi-feature objects. This method consists in determining the distance of each object from the pattern and anti-pattern of development and then their linear arrangement of objects. A similar ordering method was developed by Hellwig (1968)
